# Simulation of Code Spectrum and Code Flow of Cultured Neuronal Networks

**DOI:** 10.1155/2016/7186092

**Published:** 2016-04-27

**Authors:** Shinichi Tamura, Yoshi Nishitani, Chie Hosokawa, Tomomitsu Miyoshi, Hajime Sawai

**Affiliations:** ^1^NBL Technovator Co., Ltd., 631 Shindachimakino, Sennan 590-0522, Japan; ^2^Department of Radiology, Graduate School of Medicine, Osaka University, Suita 565-0871, Japan; ^3^Biomedical Research Institute, AIST, Ikeda, Osaka 563-8577, Japan; ^4^Department of Integrative Physiology, Graduate School of Medicine, Osaka University, Suita 565-0871, Japan; ^5^College of Health and Human Sciences, Osaka Prefecture University, Habikino, Osaka 583-8555, Japan

## Abstract

It has been shown that, in cultured neuronal networks on a multielectrode, pseudorandom-like sequences (codes) are detected, and they flow with some spatial decay constant. Each cultured neuronal network is characterized by a specific spectrum curve. That is, we may consider the spectrum curve as a “signature” of its associated neuronal network that is dependent on the characteristics of neurons and network configuration, including the weight distribution. In the present study, we used an integrate-and-fire model of neurons with intrinsic and instantaneous fluctuations of characteristics for performing a simulation of a code spectrum from multielectrodes on a 2D mesh neural network. We showed that it is possible to estimate the characteristics of neurons such as the distribution of number of neurons around each electrode and their refractory periods. Although this process is a reverse problem and theoretically the solutions are not sufficiently guaranteed, the parameters seem to be consistent with those of neurons. That is, the proposed neural network model may adequately reflect the behavior of a cultured neuronal network. Furthermore, such prospect is discussed that code analysis will provide a base of communication within a neural network that will also create a base of natural intelligence.

## 1. Introduction

Many generator models and analyzing methods of spike trains from neurons including artificial models, such as spike-coding metrics [1], spatiotemporal coding models [2–8], synchronous action models [9–13], and pseudorandom code detection methods [14] have been proposed.

**Figure 1:**
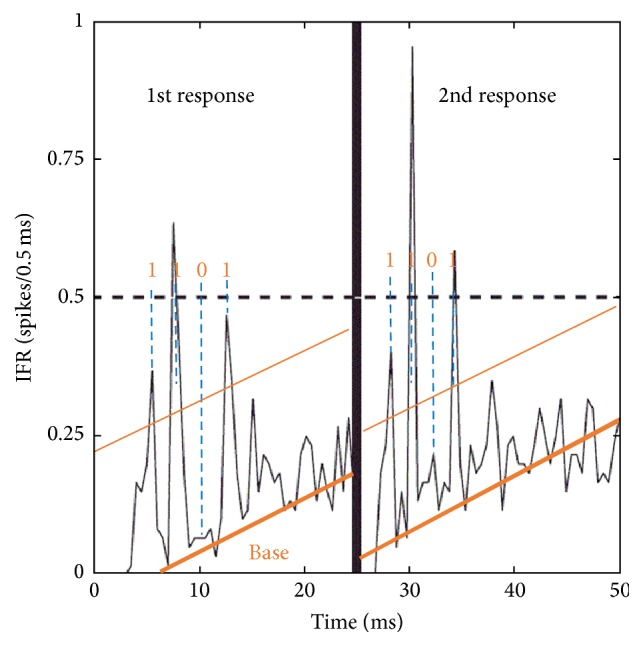
First and second responses “1101” in the instantaneous firing rate (IFR) of cultured neuronal tissue after two electrical stimulations as shown in [15] with additional interpretation (courtesy of Baljon et al.; reproduction permission provided by APS).

**Figure 2:**
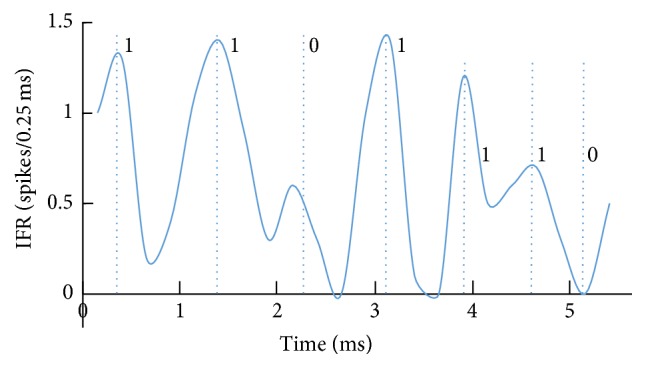
Instantaneous firing rate (poststimulus time histogram) where the sequence “1101” was observed. Since the timing becomes dispersed within trials, the peak positions and shapes gradually changed as time elapsed. Particularly, there was a tendency of risings and peaks to become faster than the regular timing, which is led by the fastest spike among the dispersed spikes as well as the slowest spike to be cancelled by the succeeding fastest negative spike (effectively supposed; pulling down the tail of peak), and lowering the peak height [16, 17].

On the other hand, we identified the sequence “1101” within the poststimulus time histogram (PSTH)/instantaneous firing rate (IFR) of a cultured neuronal network in Baljon et al. [15] (Figure 1) as well as our own network [16, 17] (Figure 2). The sequence “1101” is the core portion of the reversal M-sequence “1101000” which is a typical pseudorandom sequence [18–21]. Electric circuits generating pseudorandom sequences are well known. Furthermore, it has been shown that, in a cultured neuronal network on a multielectrode, pseudorandom-like sequences (codes), including “1101”, are detected, and they flow with some spatial decay curve [22]. We call the chart of the appearance frequencies of short code (e.g., length <8) versus various codes (in number) “code spectrum.” Each cultured neuronal network is characterized by a specific code spectrum curve. That is, the curve shape seems to depend on the characteristics of neurons and the network configuration, including the weight distribution in the network. Therefore, we may consider it as a “signature” of the network. In the present study, we performed a simulation of code spectrum from a multielectrode on a 2D mesh neural network using neurons with fluctuating characteristics of refractory period and output delay time around each given intrinsic characteristic from time to time; that is, parameters of each neuron varied intrinsically and temporarily. Connection weights between neurons are randomly generated on the basis of a given statistical property. In this paper, expression “neuronal network” is mainly used for natural one, and “neural network” is for artificial one.

## 2. Simulation

### 2.1. Network Configuration

**Figure 3:**
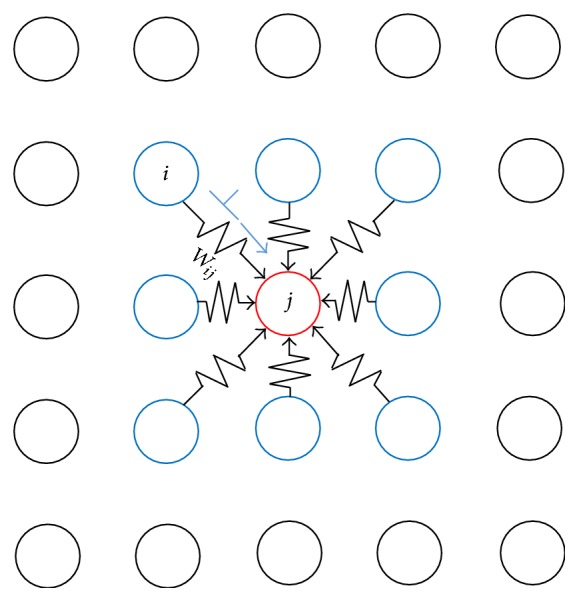
Inputs to neuron *j* in a 2D mesh neural network. Each neuron *j* (red) receives input spikes from eight neighboring neurons (blue) such as *i* through connection weights *w*_*ij*_ ∈ [−1,1]. Neuron *j* integrates such weighted spikes during its accepting period and outputs a spike after a delay time if the integrated value exceeds zero.

We performed a computer simulation to observe spike propagation on a 2D network with a 33 × 33 2D mesh of neurons. Each neuron has connection weights to and from eight neighboring neurons, as shown in Figure 3.

The weight of the network is given as follows:

(1) $$\begin{array}{c} w_{ij}=F\left[\;\left(\;1+c\;\right)u-c\;\right],\;w_{ij}\in \left[-1,1\right],\;\;w_{ij}≢w_{ji}, \end{array}$$

where *i* = (*i*_*x*_, *i*_*y*_); *j* = (*j*_*x*_, *j*_*y*_); *i*_*x*_, *i*_*y*_, *j*_*x*_, *j*_*y*_ ∈ {1,2,…, 33}; *i* ≠ *j*; max{|*i*_*x*_ − *j*_*x*_|, |*i*_*y*_ − *j*_*y*_|} = 1; and *F*[*x*] is a truncation function such that

(2) Fx=11≤x=x−1<x<1=−1x≤−1,

*c* is positive parameter such that 0 < *c* ≤ 3 controls weight balance of positive and negative values and *u* is a random variable with uniform distribution such that 0 ≤ *u* < 1.

In short, if *c* increases, the number of negative weights increases. For example, if *c* = 1/3, weight *w*_*ij*_ distributes uniformly in [−1/3, 1]; that is, the ratio of positive to negative weights is 3 : 1. This ratio is a typical one in real neuronal networks. In 2D mesh artificial neural network model with weights to and from every eight neighboring neurons, however, weight distribution must be different because of different network shape and every eight neighboring weight is generated randomly, which usually does not result in zero. Of course, such models can be designed that first decide whether or not the weight exists and then decide on the weight value if it exists. For simplicity, we have designed that no connections between adjacent neurons (i.e., not connected; weight zero) are effectively realized by suppressions with negative weights. If *c* = 1, *w*_*ij*_ distributes in [−1,1] uniformly; that is, the ratio of positive to negative weights is 1 : 1. If *c* = 3, *w*_*ij*_ distributes in [−1,1] but not uniformly; that is, the ratio of positive to negative weights is 1 : 4. Although the setting of weights was simple, the result was not much different from that obtained using separate settings of the weight values and their positive or negative signs.

### 2.2. Characteristics of Neurons

**Figure 4:**
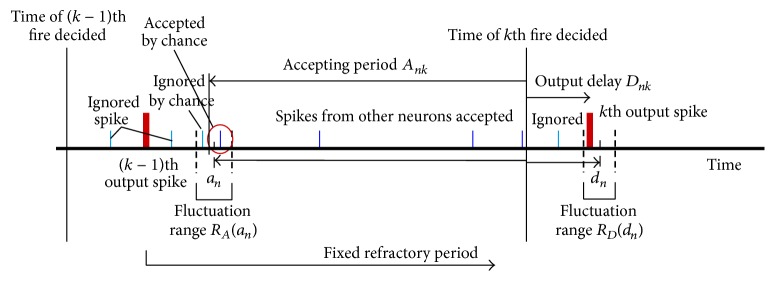
Integrate-and-fire model without leakage but with fluctuation in the parameters of neuron *n*. Each neuron has an inherent accepting period *a*_*n*_ and output delay time *d*_*n*_. These parameters vary with time within certain ranges *R*_*A*_(*a*_*n*_) and *R*_*D*_(*d*_*n*_), respectively. Neuron *n* integrates weighted input spikes during the accepting period *A*_*nk*_ for the *k*th firing, and after the refractory period ends, it decides whether the integrated value exceeds zero for firing at every time point. If so, it outputs the *k*th output spike with delay time *D*_*nk*_. That is, *A*_*nk*_ ∈ *R*_*A*_(*a*_*n*_), and likewise, *D*_*nk*_ ∈ *R*_*D*_(*d*_*n*_).

The neuron model used here is shown in Figure 4, which is a type of integrate-and-fire model without leak [25]. Neuron *n* accumulates weighted inputs during the accepting period *A*_*nk*_. If the accumulated weighted input becomes positive and the neuron is not in its refractory period, the neuron *n* outputs a spike after a delay time *D*_*nk*_ as the *k*th firing. Some parameters used in the simulation are given as examples below.

A basic accepting period *a*_*n*_ intrinsic to neuron *n* is randomly generated in each network within a range *a*_*n*_ ∈ {*a*_0_ − 2, *a*_0_ − 1, *a*_0_, *a*_0_ + 1, *a*_0_ + 2} with a probability of 1/5 for each, where *a*_0_ is a fixed common parameter value through the network, and the unit time (bin) is 0.1 ms. The actual instantaneous accepting period *A*_*nk*_ of neuron *n* at the *k*th firing is further randomly given within the fluctuation range *R*_*A*_(*a*_*n*_) around *a*_*n*_; that is, *A*_*nk*_ ∈ *R*_*A*_(*a*_*n*_). The instantaneous refractory period is implicitly assumed as slightly smaller than *A*_*nk*_. The accepting period can also be called input integration or the accumulating period for firing. Roughly speaking, the accepting and refractory periods can be regarded as the same. The software can stimulate neurons with arbitrary spatiotemporal patterns. Typically, a combination of three neurons is simultaneously stimulated such that the states of the neurons are set to “1” at time 1 (bin no. 1). The reason why we stimulated three neurons simultaneously instead of only one neuron is that, in wet experiments, many neurons around an electrode seem to be simultaneously stimulated by an electrical stimulation; and secondly, parallel stimulation to multiple neurons seems to promote stable propagation of excitation according to computer simulations.

Parameters used in the simulation are as follows.

Basic accepting period *a*_0_ is a preassigned network parameter such as 20,30,…, 100, where unit time (bin width) is 0.1 ms. Instantaneous accepting period *A*_*nk*_ ∈ *R*_*A*_(*a*_*n*_) = {*a*_*n*_ − 1, *a*_*n*_, *a*_*n*_ + 1} is randomly selected at the *k*th firing with the probability *p*_*a*_, 1 − 2 × *p*_*a*_, and *p*_*a*_, respectively, and *p*_*a*_ = 1/12. This value defines how much the instantaneous accepting period fluctuates from time to time. If we increase *p*_*a*_ or expand the range *R*_*A*_(*a*_*n*_), the fidelity of communication or information flow decreases. The above value is determined by considering a trade-off between such considerations and attaining 99% communication fidelity. Details will be presented in our coming paper.

Similarly, the output delay is as follows.

Basic output delay time *d*_*n*_ is randomly selected from {2,3,…, 8} with a probability of 1/7 each. Instantaneous output delay time *D*_*nk*_ ∈ *R*_*D*_(*d*_*n*_) = {*d*_*n*_ − 1, *d*_*n*_, *d*_*n*_ + 1}, and *p*_*d*_ = 1/12, similar to *R*_*A*_(*a*_*n*_).

We have utilized an integrate-and-fire model without leaks, but instead we used a model incorporating a fluctuating period of acceptance. Our model is more stringent than the leaky integrate-and-fire model because our model randomly neglects old spikes, while the leaky model neglects them gradually. However, from our simulations, ignoring a portion of the spikes in any type is not fatal and not essential but only influences the quality of communication. This property is a strong point of neural networks that are composed of essentially fluctuating neurons. The fluctuating time parameters are concentrated in the two parameters—the accepting period, preceding the fire deciding time and the output delay time, following this time. These designs were determined by balancing the degree of complication, essential precision of results, and computation time.

**Figure 5:**
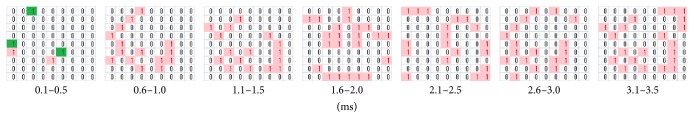
Spike waves generated on a 2D mesh neural network. Green spots indicate stimulation point. “0.1–0.5 ms” means an accumulation result of firing at 0.1 ms, 0.2 ms,…, 0.5 ms. This suggests that the codes are a part of these “spike waves.”

Results of a preliminary experiment of stimulating a 9 × 9 mesh neural network are shown in Figure 5, where three neurons were stimulated at time *t* = 0. We can observe “spike waves” propagating from source stimulations, and the waves often behave like Synfire [9, 10]. Further, the code flow may be regarded as a component of such spike waves. It is possible to acquire codes in wet experiments and compare them with the simulation. In the present study, however, as a preliminary step, we focused on simulating not only the entire spike waves but also the flow of codes.

### 2.3. Arrangement of Multielectrode

**Figure 6:**
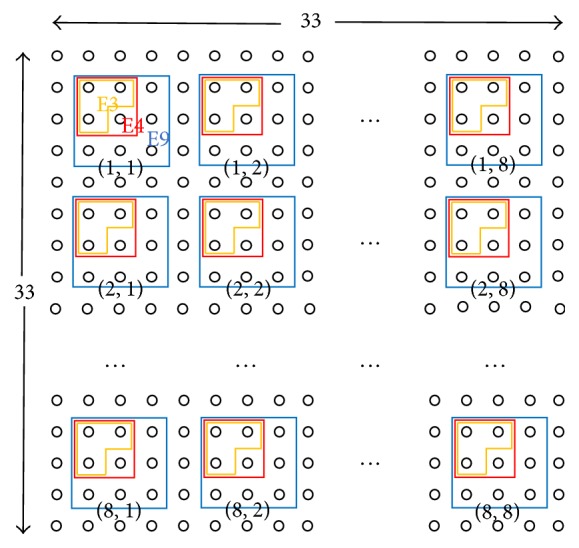
Arrangement of 8 × 8 multi-electrodes [(1,1), (1,2),…, (8,8)] on a simulated 33 × 33 2D mesh neural network. Each electrode acquires spikes of two to nine neurons. For example, “E9” showing 3 × 3 block of neurons (○) indicated with (1,1) shows that electrode (1,1) collects spikes from nine neurons. In addition, “E3” and “E4” are likewise; “E2” and “E5”–“E8”are not shown. Spectrum, cross-correlation, or probability distribution data obtained from electrode Em is expressed as “*E*_*m*_” in the text. Connections between eight neighboring neurons are randomly generated with given stochastic characteristics.

Figure 6 shows the arrangement of a multielectrode on a 33 × 33 2D mesh neural network. Each neuron has directional connections with eight neighboring neurons. Each electrode gathers spikes from two to nine surrounding neurons. The major differences from the wet experimental configuration [22] are as follows: (1) there are no neurons and no connections outside the explicit 1089 neurons, (2) neurons are placed on a regular lattice, which is different from an irregularly shaped cultured neuronal network, and (3) distances between electrodes are smaller than those in the cultured neuronal network. It can be said that difference (3) is a smaller scale of (1) and it corresponds to the fact that we regard the spike data from different electrodes as independent events. That is, in (1) and (3), we neglect the behavior of neurons outside the noted neurons.

### 2.4. Component Code Spectrum

**Figure 7:**
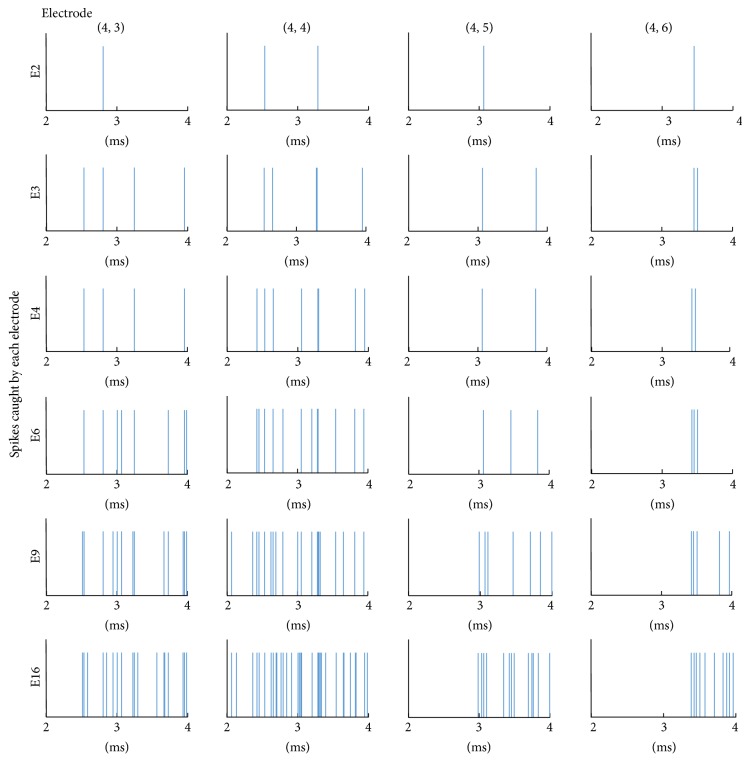
An example of simulated spike trains caught by Em during 2.0–4.0 ms after stimulation with *a*_0_ = 8.0 ms and *c* = 2.5. Initial stimulations were given to around electrodes (1,1) at 0.1 ms and (1,5) at 0.5 ms.

By changing the basic accepting period *a*_0_ and the positive and negative weight balance parameter *c*, we can generate spike trains for each type of electrode Em catching from *m*  (*m* = 2,3,…, 9) surrounding neurons. We then decode the spike trains to obtain code spectra for each *m*. We call such a spectrum *E*_*m*_ “component spectrum.” Figure 7 shows an example of simulated spike trains caught by electrode Em.

**Figure 8:**
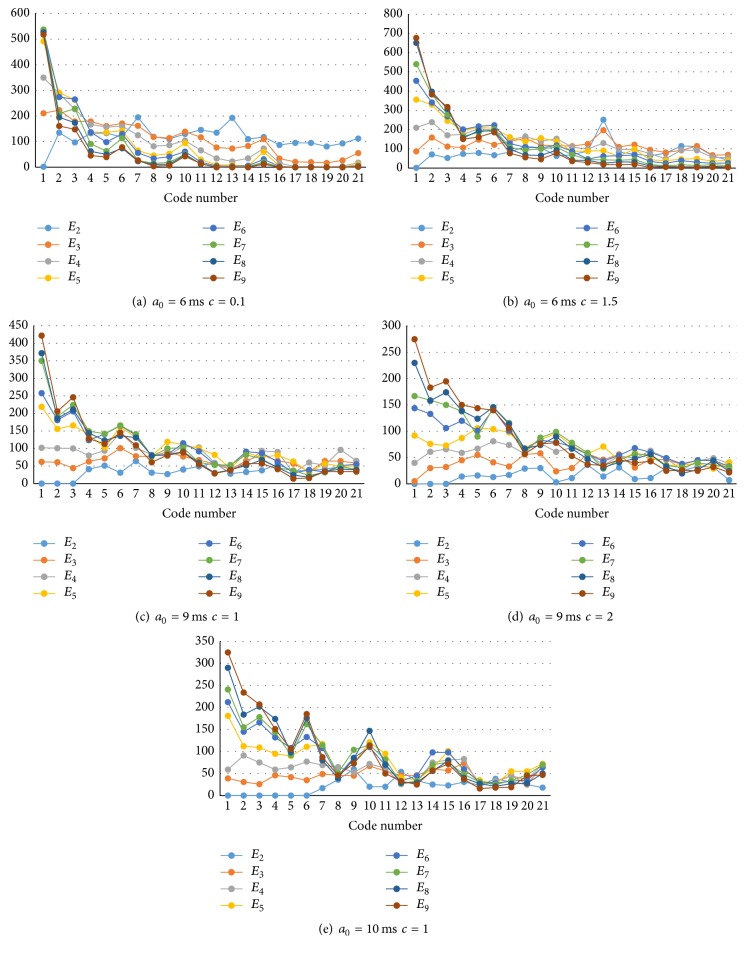
Code spectrum components *E*_*m*_ for several parameters of accepting period *a*_0_ and positive and negative weight balance *c*. The horizontal line represents code numbers (1,2,…, 21) whose number of “1”s in the code is 3. That is, code 1 = “111,” code 2 = “1011,” code 3 = “1101,” code 4 = “10011,” code 5 = “10101,” … code 21 = “11000001” [22]. The vertical line represents the total number of codes detected during the first 200 ms after stimulation (2000 time bins of 0.1 ms/bin) and from 63 electrodes.

Figure 8 shows examples of component code spectra of simulated spike sequences of some *a*_0_ (median of accepting period *A*_*nk*_) and the connection weight parameter *c*. Practically, the number of neurons emitting spikes to each electrode changes electrode by electrode according to its statistical distribution. Therefore, the actual observed code spectra will be mixed according to the probability distribution of the number of neurons *m* around the electrodes.

### 2.5. Spectrum Fitting with Components

**Figure 9:**
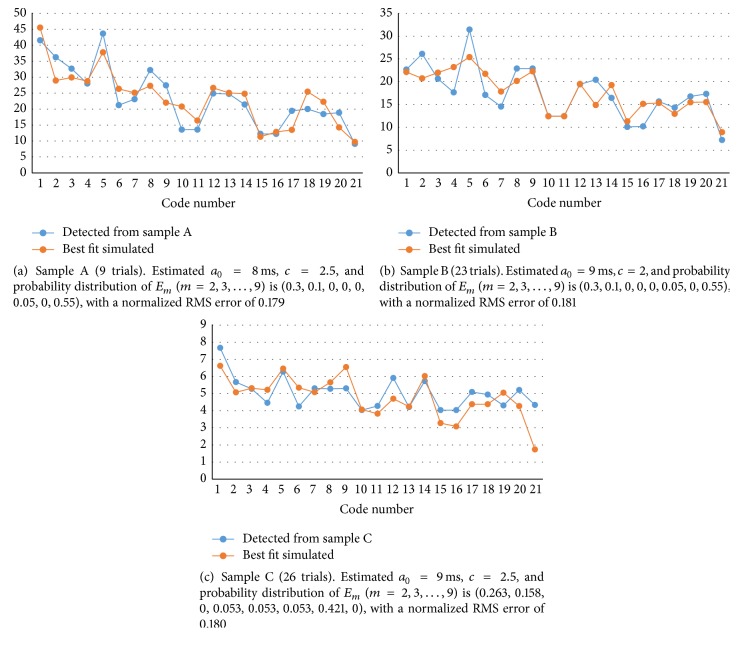
Code spectra. The blue curve shows the average of trials of an experimental number of codes detected from 63 electrodes in spike trains during 200 ms after stimulation expressed with 2000 time bins of 0.1 ms. The bit width of the code is 0.6–2.0 ms (6–20 bins). Codes are detected with 1% time accuracy, although practically several % because of the 0.1 ms bit width. This curve can be considered as the “signature” of spike trains. The orange curve is the best fit to code spectrum using a simulation spectrum.

The blue curve in Figure 9(a) shows the results of the average number of codes detected in recorded spike trains of 9 trials for code numbers 1–21 (code spectrum) from 63 electrodes of Sample A during 200 ms after stimulation expressed as 2000 times the bin data. Sample A is the same as that presented in [22].

The orange curve in Figure 9(a) shows the best fit to the number of codes detected in spike trains of 2000 time bins of Sample A using code spectra *E*_*m*_ of artificially generated spike sequences with different accepting periods *a*_0_ and connection weight parameters *c* and probability distribution of *E*_*m*_. Although this is an inverse problem and imperfect, the estimation of neuronal parameters *a*_0_, *c*, and probability distribution of *E*_*m*_ is possible to some extent.

Table 1 shows the normalized squared error of a simulated code spectrum to that of recorded data of Sample A with a changing probability distribution of *E*_*m*_, that is, a combination of component code spectra for various *a*_0_ and *c* values. Some root mean square (RMS) error data are not shown (shown as “—”) because the number of codes detected was too small to calculate the component code spectrum as such spikes disappeared within the given time. Tables 2 and 3 show that of Sample B in Figure 9(b) and Sample C in Figure 9(c), respectively.

### 2.6. Expansion of *E*_*m*_

**Figure 10:**
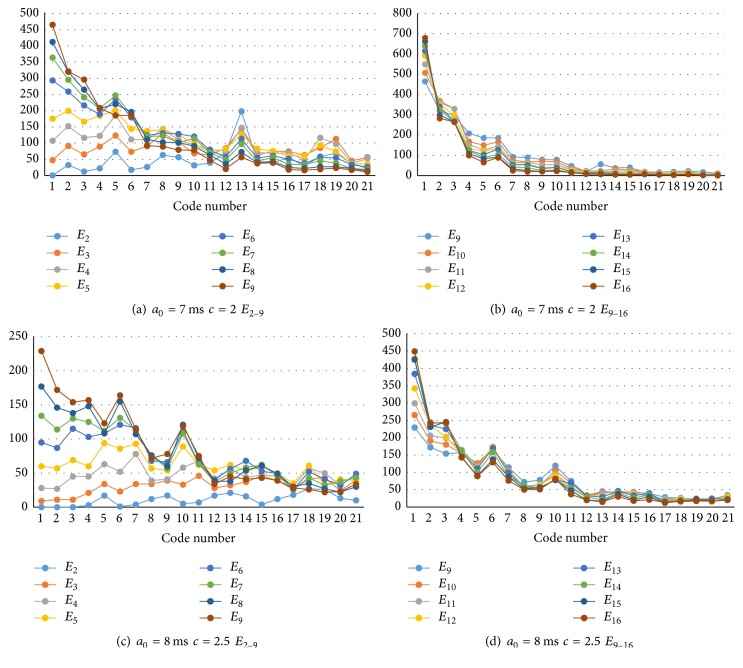
Expanded spectrum components up to *E*_16_. (a) Code spectrum components *E*_2_–*E*_9_ for *a*_0_ = 7 ms and *c* = 2. (b) Expansion to *E*_9_–*E*_16_ of (a). (c) Code spectrum components *E*_2_–*E*_9_ for *a*_0_ = 8 ms and *c* = 2.5. (d) Expansion to *E*_9_–*E*_16_ of (c).

In some cases, the best estimation of the probability distribution of *E*_*m*_ had a large value at *E*_9_, suggesting that there are more than nine neurons around the electrode. Therefore, we increased the number *m* to 16.  Figure 10 shows the expanded code spectral components up to *E*_16_. Although *E*_2_–*E*_9_ have various shapes, *E*_9_–*E*_16_ have a similar shape. When the best fit process included a large number of parameters, the computation time was long. Therefore, it may be reasonable to regard *E*_9_ as the representative component of *E*_9_–*E*_16_ to decrease the computation time.

### 2.7. Maximum Cross-Correlations

**Figure 11:**
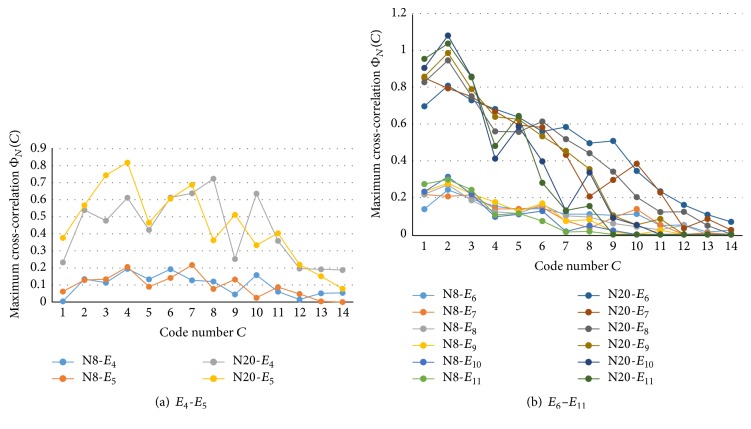
Maximum cross-correlation Φ_*N*_(*C*) of a trial among eight (8N) and 20 (20N) neighbors with a time frame difference of 0.5 ms for 14 major codes *C*.

Because the simulation size was limited, the separation distances between the electrodes were very small (1–4 times of the neuron pitch). Nevertheless, using the same method as used in [22] for the natural neuronal network, we calculated the maximum cross-correlation Φ_*N*_(*C*) of a trial among eight and 20 neighbors between a time frame difference of 0.5 (ms) for *E*_2_–*E*_16_ and 14 major codes *C* (Figure 11). However, since the electrodes were located relatively closer than in [22], and therefore cooccurrence probability of each code between two electrodes was expected higher, normalization of cross-correlation by code length was not applied. As shown in Figure 11(a), in case of *E*_4_ and *E*_5_, the maximum cross-correlation is rather flat in comparison with that of Sample A of the natural neuronal network of [22]. The characteristics of *E*_6_–*E*_11_ (Figure 11(b)) decreased according to the code number, which roughly represents the code length. Although not shown in figures, *E*_12_–*E*_16_ showed a more steep decreasing tendency.

## 3. Discussion and Conclusions

In the present study, we constructed a 2D mesh neural network model and an input/output (I/O) functional model of neurons. A 2D mesh model may differ from a real natural cultured neuronal network, which will have a nonhomogeneous distribution of neurons [26]. The I/O functional model of neurons has certain intrinsic and instantaneous fluctuations of the refractory period and output delay time [11]. There may be some discrepancies compared with real natural cultured neurons; however, we have shown that from the perspective of the code spectrum of spike trains, it is possible to simulate the electrical activity of a neuronal network on a multielectrode plate (MED plate) with a 2D mesh neural network model. Moreover, it was possible to estimate the neuronal network parameters to some extent by recording spike trains with electrode without sorting. For example, though there is no guarantee to be true ones, estimated typical accepting periods were 8-9 ms, and refractory periods were also estimated as almost the same which are within range (5–10 ms) of a text book [27].

The detected codes were composed of spikes from different neurons because we acquired codes with bit widths ranging from 0.6 to 2.0 ms; in cases of 4-bit code, code lengths are mainly less than 6 ms (= 2 ms × 3 [spans of 4-bit code]). These values are generally shorter than the refractory period of neurons, which is estimated to be more than 8 ms in our cases. This suggests that it is unnecessary to add sorting processing to the spikes obtained from each electrode in case of analyzing by code with length less than 8 ms. That is, spikes forming the codes under our conditions originate from different neurons, and it may be possible to analyze circuit states around the electrodes. We need not to deal with *E*_1_ whose spike interval is more than 8 ms. In our case, although we statistically analyzed all the 63 electrodes (except for the stimulating electrode) together, it may be possible to analyze individual electrodes, which may give more precise distribution of *E*_*m*_; that is, we can determine whether neurons near a certain electrode compose a specific circuit such as a linear feedback shift register (LFSR) [14].

We used simulated code spectrum components to analyze the wet experimental spectrum of a cultured neuronal network. It is true, however, there are problems of (1) besides codes are not always orthogonal, (2) some of the extracted codes may be false such that composed of overlapped spike combination by chance [22]. To reduce such effects, it may be effective to improve the statistical background such as increasing the number of spike trains to suppress the statistical variation of the code spectrum components, because the present data were acquired from only one spike train of one trial each, that is, from the 63 electrodes, which are not necessarily perfectly independent.

A 2D mesh neural network can generate spike waves, as shown in Figure 4. Thus, the code flow observed on a cultured neuronal network can be regarded as a fragment of “spike waves.” Code not only works as a marker of the spike waves but also provides information or clues about the circuit's shape. In other words, the code spectrum reflects the circuit shape, including the weight distribution, neuron characteristics, and its role in communication around each electrode. Therefore, it may be considered as a kind of network signature.

**Figure 12:**
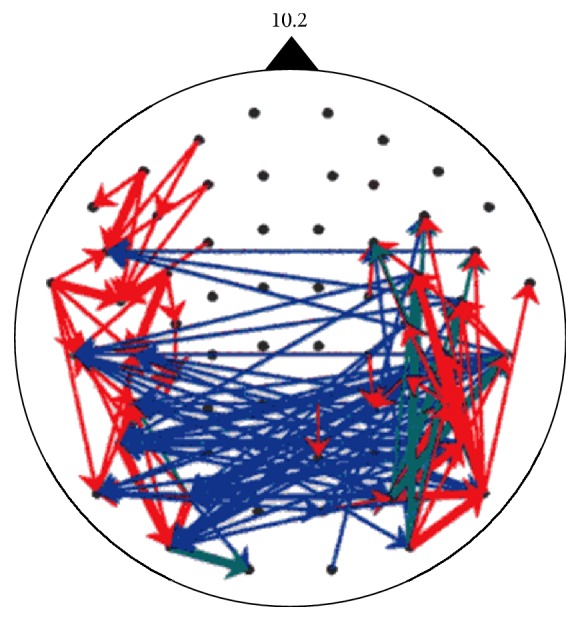
Time-shift diagram of 10.2 Hz MEG, for a number counting task [23, 24]. Red arrow < 5 ms < green < 10 ms < blue. We can see that red arrow with lag time < 5 ms runs within each hemisphere, and blue > 10 ms across the callosum.

**Figure 13:**
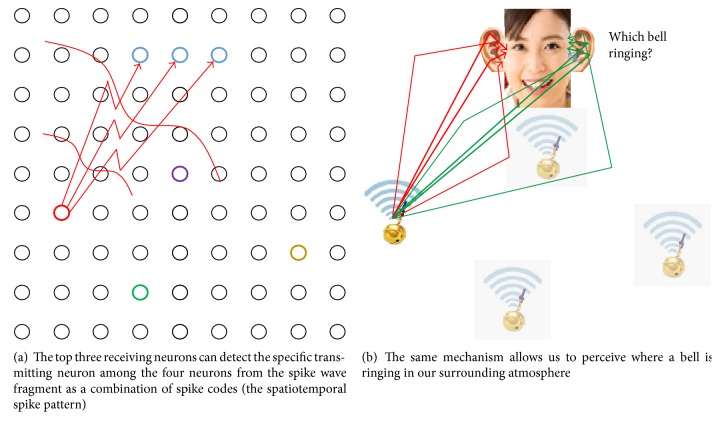
Illustration of the communication within the neural network based on spatiotemporal pattern recognition. Each neuron can perform the roles of transmitting, receiving, and simply as a transmission media.

Furthermore, spike waves will establish asynchronous multiplex communication links as well as multiplex communication within a synchronous neural network [23, 28] where various pseudorandom-like codes are observed. For communication within this network, each neuron or group of neurons cannot receive entire spike waves, but rather a specific part of the wave, that is, a pseudorandom-like code or a spatiotemporal combination of codes. Then, based on the communication links as presented in Figure 12 [24, 29], information will be processed by an intelligence mechanism in the brain. Under these conditions, information will be radiated as spike waves from source neurons and then widely propagated via the neuronal network. There has been substantial research on behavior of a spike intensity wave as a global macrotask including cardiac electric propagation [30–32]. Although it may be true that most neurons work to relay spikes as a part of spike waves, destination neurons in a communication task are limited in number and must select signals sent to them from among the various spike waves based on spike trains containing codes that are a fragment of each spike wave or a spatiotemporal combination of these codes. This task is local and should not be treated as conventional “intensity” level wave information, but rather as “locally phasic” level wave information incorporated in the spatiotemporal pattern of locally arriving spikes including codes. In artificial brain research, though through concentrated digital synaptic switching, effect of communication is shown [33]. The present study serves a base of communication tasks in natural intelligence, as illustrated in Figure 13. These studies of spike waves will lead to higher-order reasoning/intelligence in the brain via communication [8, 34].

Our results, including those reported in [22], are summarized as follows:

1. We are investigating an intelligence mechanism with the maintenance of correspondence between wet experiments and simulation.
2. We identified a pseudorandom sequence (code) “1101” for PSTH in the literature as well as from our experience which should provide clues about neuronal circuits around electrodes.
3. A code spectrum as an extension of code “1101” was obtained from cultured neuronal networks and simulations.
4. A code spectrum can be considered as a “signature” of its associated network by which some characteristics can be estimated, including the refractory period and weight distribution.
5. A code can function as a mark on a spike wave. We have shown that to some extent, wave propagation preserves the codes. This finding was documented by movies as well as quantitatively.

Future studies will address the following:

- communication based on codes as a part of spike waves,
- organization of communication links to derive intelligence functions.

## Figures and Tables

**Table 1:** Normalized RMS error for various parameters of neurons used to fit the data in Figure 9(a). Bold and italic  values show the first and second best fits, respectively. Typical *a*_0_ and *c* values were estimated as 8.0 ms and 2.5, respectively.

*c*	*a*_0_ (ms)					
	5.5	6.0	7.0	8.0	9.0	10.0
0.1	0.464	0.315	0.565	—	—	—
0.3	0.442	0.283	0.581	0.904	—	—
1.0	0.393	0.252	*0.182 *	0.212	0.327	0.349
1.5	0.326	0.240	0.208	0.230	0.269	0.286
2.0	0.301	0.246	0.203	0.212	0.186	0.310
2.5	—	0.470	0.580	**0.179 **	0.192	—
3.0	—	0.321	—	0.328	0.319	0.674

**Table 2:** Normalized RMS errors for various parameters of neurons used to fit the data in Figure 9(b).

*c*	*a*_0_ (ms)					
	5.5	6.0	7.0	8.0	9.0	10.0
0.1	0.502	0.322	0.577	**—**	**—**	**—**
0.3	0.484	0.288	0.577	0.890	**—**	**—**
1.0	0.438	0.259	0.197	0.233	0.335	0.340
1.5	0.361	0.243	0.210	0.248	0.271	0.283
2.0	0.320	0.256	0.188	0.241	**0.1809**	0.304
2.5	**—**	0.477	0.605	0.203	*0.1814*	**—**
3.0	**—**	0.325	**—**	0.317	0.332	0.680

**Table 3:** Normalized RMS errors for various parameters of neuron used to fit the data in Figure 9(c).

*c*	*a*_0_ (ms)					
	5.5	6.0	7.0	8.0	9.0	10.0
0.1	0.518	0.290	0.461	**—**	**—**	**—**
0.3	0.509	0.223	0.440	0.777	**—**	**—**
1.0	0.515	0.291	0.252	0.204	0.222	0.223
1.5	0.430	0.275	0.253	0.196	0.197	0.187
2.0	0.399	0.257	0.263	0.256	*0.186 *	0.215
2.5	**—**	0.491	0.656	0.213	**0.180 **	**—**
3.0	**—**	0.324	**—**	0.354	0.358	0.631
